# Distilling the distillers: examining the political activities of the Distilled Spirits Council of the United States

**DOI:** 10.1186/s12992-023-00923-y

**Published:** 2023-03-29

**Authors:** Matthew Lesch, Jim McCambridge

**Affiliations:** Department of Health Sciences, University of York, York, UK

**Keywords:** Alcohol policy, Alcohol industry, Framing, Lobbying, Global public health, Commercial determinants of health

## Abstract

**Background:**

Understanding of the alcohol industry’s means of influencing public policy is increasingly well established. Less is known, however, about the specific organisations that lead the political strategies of the alcohol industry. To fill this gap, this paper explores the Distilled Spirits Council of the United States (DISCUS), a key trade association in the United States (US), which also operates internationally.

**Methods:**

This study explores how DISCUS is organised and the main political activities it pursues to advance its policy interests. The study triangulates data from several sources, including DISCUS documents, as well as federal lobbying and election expenditure data.

**Results:**

This study demonstrates that DISCUS is a key political actor in the US and global alcohol policymaking context. There are identifiable strategies used by DISCUS to shape alcohol policy debates, including framing and lobbying. We also find key synergies between these strategies and identify their operation at varying levels of policy decision-making.

**Conclusions:**

Generating more secure inferences about the nature of the alcohol industry’s efforts to advance its interests, and with what success and at what cost, requires researchers to investigate other trade associations in different contexts, and use other data sources.

## Introduction

The alcohol industry comprises a formidable set of political actors that operate within and across several jurisdictions. Public health researchers have identified the web of actors and activities comprising the alcohol industry within certain countries [1–3]. Like other industries, trade associations have been used as the main vehicles to organise political activities [2, 3]. Trade associations (TAs) allow erstwhile economic competitors to collaborate to advance shared interests in the political sphere [4]. TAs enable individual companies to acquire information about socio-political issues and access resources which “transcend” organisational boundaries [5].

In the alcohol field, TAs tend to be organised by sector, such as spirits or beer, or role in the supply chain, such as producers or retailers [6, 7]. The Distilled Spirits Council of the United States (DISCUS), Brewers of Europe, and the British Beer and Pub Association are notable examples [8]. A key function is to represent interests in both domestic and international political processes [9], allowing alcohol companies to “speak with a single voice” on matters of policy [3, 7]. Increased concentration of ownership within the sector has made such coordination easier [10, 11]. Although there are some exceptions [3, 12, 13], despite the importance of alcohol TAs at the global and domestic level, these organisations have largely escaped dedicated study. There are sound reasons to study the operation of these organisations. In other contexts, including the tobacco, asbestos and chemicals industries, in-depth analyses of TAs and their activities have demonstrated how they serve as vehicles for protecting corporate interests in the face of regulatory or political pressure [14–18]. When it comes to alcohol, there is currently insufficient understanding of the various functions of TAs in different political contexts, as well as the extent of their activities.

DISCUS is an association representing the distilled spirits supply chain including producers, marketers, and exporters. It is one of the leading alcohol TAs in the US and for the spirits sector, DISCUS plays a key role in monitoring relevant policy concerns across varying levels of governance [6]. For example, in the 1980s, DISCUS partnered with tobacco interests and other allies to mobilise against attempts to raise federal and state excise taxes [19, 20]. DISCUS has also been active in influencing international trade and global health policy agendas [21, 22]. DISCUS has also been identified as a key player in other attempts to shape alcohol policy and science [6, 7, 23–25]. Notwithstanding these studies, there is limited understanding of how DISCUS operates and the tools it uses to engage in the political process.

This paper offers thick description and analysis of DISCUS and its main political activities [26]. It aims to provide a preliminary case study of alcohol TAs as political actors. Such an analysis has the potential to enrich understanding of how what could be a key actor operates in the US and international context. Moreover, the study has the potential to offer broader insights into the roles of TAs in shaping national and global alcohol policy developments.

## Methods

We first performed searches of DISCUS’s website as well as conducted additional web searches using Google for orientation purposes. We then collected documents from DISCUS’s website and supplemented them with documents and data from several websites, including the World Health Organization (WHO), OpenSecrets, and the Secretary of the Senate’s Office of Public Records (SOPR). The key data sources are described in turn and summarised in Table 1.

Documents collected from DISCUS’s website took the form of public-facing materials such as press releases (n = 85) and correspondence to lawmakers (n = 9). In other instances, we identified presentations and briefing materials (n = 9) which appeared to be produced for DISCUS members or other stakeholder audiences. This second category of documents is particularly interesting as it has the potential to offer insights into the internal dynamics of DISCUS, particularly in respect of how it defines and communicates its goals, priorities, and activities to its members. We collected further documents detailing other aspects of the organisation’s activities (n = 7), including fact sheets and campaign materials. Finally, from the WHO website, we retrieved DISCUS’s consultation submission (n = 1) to the WHO’s Global strategy to reduce the harmful use of alcohol.

OpenSecrets is a website that provides several searchable databases on campaign contributions and lobbying and links to documents such as Federal Election Commission (FEC) filings and lobbying records. OpenSecrets has been used previously by researchers to address similar research questions [27, 28]. In the US, interest groups that engage in lobbying or make campaign contributions to candidates or political action committees (PACs) are required to make disclosures to relevant oversight bodies. From the OpenSecrets website, we used keyword searches in its lobbying expenditure and lobbying activities databases, searching for “Distilled Spirits Council.” From this search, we collected several records which detailed DISCUS’s lobbying expenditures (n = 23) as well as the main agencies (n = 23) and policy issues (n = 22) that the lobbying focused on. These records covered DISCUS’s activities between 1998 and 2021.

To provide a closer look at lobbying activities, we examined some of the main source material that OpenSecrets uses to furnish its database. In the US, all lobbying disclosures must be submitted to the Secretary of the Senate’s Office of Public Records (SOPR), while any financial contributions to candidates must be declared to the FEC. Under the Lobbying Disclosure Act of 1995, federal lobbyists are required to disclose clients they represent, income generated from lobbying, and the specific policy issue they are lobbying about. These records are fully accessible through the SOPR and FEC websites. For this analysis, we were particularly interested in the lobbying records that OpenSecrets referenced. We used keyword searches in the SOPR’s database (e.g., “Distilled Spirits Council”). This returned a total of 84 individual lobbying reports for DISCUS. Each report provides a quarterly report of lobbying activities, including how much money was spent on lobbying and the specific policy issues that were lobbied on (e.g., the WHO’s global strategy to reduce harmful use of alcohol). We used these records to get an overall sense of the main types of policy issues that DISCUS lobbies on and to cross-reference specific data from the OpenSecrets records. Given the scope and detail of each lobbying record in the SOPR database, a comprehensive analysis of each record was beyond the scope of this study (see limitations in Discussion).

Documents were placed into NVivo by the first author. To analyse the content of the documents, we formulated thematic codes [29]. Our approach to coding and analysis was informed by previous research on the commercial determinants of health [30, 31], corporate power, and the alcohol industry specifically [32, 33]. We developed a broad set of codes to identify the type of political strategy (e.g., issue framing, lobbying, coalition-building, and political donations). We also developed codes to capture relationships or synergies between these strategies. Finally, we allowed new themes to develop, particularly in better understanding the context of these strategies. For example, in analysing DISCUS-produced materials, we coded for the specific policy issues that were discussed (e.g., taxes, restrictions on availability) as well as the institutional context of these documents (e.g., state, federal, and global). The analytical approach afforded a much clearer understanding of the range of DISCUS’s political activities and whether different settings (e.g., level of government, public vs. semi-public audience) were associated with different or overlapping industry strategies.

**Table 1:** Summary of data sources

Type of document	Source of material	Number of records/ documents retrieved	Method of retrieval
DISCUS press releases^1^	DISCUS website	85	https://www.distilledspirits.org/news-releases/
DISCUS correspondence to lawmakers^2^	DISCUS website	9	https://www.distilledspirits.org/ Google keyword searches (“Distilled Spirits Council and filetype:pdf”)
DISCUS presentation materials to membership^3^	DISCUS website	9	https://www.distilledspirits.org/ Google keyword searches (“Distilled Spirits Council and filetype:pdf”)
Other DISCUS website materials (e.g., fact sheets, campaigns materials)^4^	DISCUS website	7	https://www.distilledspirits.org Google key word searches (“Distilled Spirits Council and filetype:pdf”)
Submissions to the WHO’s Global strategy to reduce the harmful use of alcohol^5^	WHO website	1	https://www.who.int/news-room/articles-detail/global-action-plan-to-reduce-the-harmful-use-of-alcohol
DISCUS federal lobbying expenditure data^5^	OpenSecrets	23	https://www.opensecrets.org/federal-lobbying/clients/summary?cycle=2022&id=D000000539
DISCUS federal lobbying activities (issues lobbied)^6^	OpenSecrets website	23	https://www.opensecrets.org/federal-lobbying/clients/issues?cycle=2022&id=D000000539
DISCUS federal lobbying activities (agencies lobbied)^7^	OpenSecrets website	22	https://www.opensecrets.org/federal-lobbying/clients/agencies?cycle=2021&id=D000000539
Lobbying disclosure reports filed with the Senate’s Office of Public Records^8^	Secretary of the Senate’s Office of Public Records (SOPR) website	84	https://lda.senate.gov/filings/public/filing/search/

## Results

We begin by providing some context for more advanced study by examining DISCUS’s self-presentation of its goals and structure. We then go on to investigate the main activities that DISCUS engages in, organising the analysis into framing and lobbying, two well-established alcohol industry political strategies.

### Self-presentation of goals, contexts and Organisational structure

DISCUS presents itself as the leading voice for distillers “on policy and legislative issues in [Washington DC], state capitals and foreign capitals worldwide”[34]. Three main activities underpin DISCUS’s approach to alcohol policy. First, DISCUS advocates for any legislative, regulatory and public affairs issues at the state, federal and international level that affect its members. This means working to “ensure the spirits sector is leading the discussion and setting the agenda with public officials and regulators.” Second, it actively promotes the sector by “raising awareness and opening markets in the United States and around the globe.” Third, it promotes “moderate and responsible consumption of distilled spirits” through a range of “evidence-based” approaches to alcohol policy [35]. DISCUS has a conventional departmental structure and employs just under 50 full-time staff [36].

DISCUS was formed in 1973 following the merger of three predecessor organisations. Today, DISCUS principal members comprise several leading spirits producers and marketers, specifically Bacardi, Beam Suntory, Brown-Forman, Campari Group, Cie, Constellation Brands, Diageo, Edrington, Hotaling & Co, Jagermeister, MGP, MHW, Moet Hennessy, Ole Smoky, Pernod Ricard, Remy Cointreau, and William Grant & Sons. In addition, there are Craft Member and Partner Member categories. According to DISCUS, the former category was launched in 2010 to better incorporate the views of small- and medium-sized distilleries. Later, in 2019 the Partner Member category was created to mobilise “the distilled spirits supply chain and related businesses.” The latter helped connect different parts of the spirits industry which are not necessarily directly involved in production, sale or marketing, but whose interests could be served through coordination and information-sharing [37].

DISCUS members have key incentives to maintain membership, including a weekly newsletter, updates on “state legislative and tax issues” and access to a website outlining laws and regulations relating to the sale and distribution of spirits across the US [38]. Companies may benefit from a TA’s lobbying efforts without incurring the direct costs of membership (i.e., free riding) [39]. DISCUS provides individual benefits for paying members. For its principal members, this allows these large multi-national companies to claim that they represent a wide membership, including small and medium businesses (see below). Within other TAs, the larger alcohol companies also tend to be dominant [3], just as they do in other sectors [5]. DISCUS seeks to portray itself to prospective members as a highly connected and politically sophisticated organisation, for example, by highlighting access to its DISCUS State Government Relations team as a membership benefit. The State Government Relations Team includes 6 vice presidents and 35 contract lobbyists that have a “track record of increasing market access and mitigating tax threats in all 50 states” [40].

Using corporate social responsibility (CSR) initiatives also represents a key strand of DISCUS’s approach to policy. In 2019 DISCUS took over the Foundation for Advancing Alcohol Responsibility (FAAR), which runs Responsibility.org. FAAR was created in 1991 as the Century Council, and since then it has been primarily funded by the major spirits companies [6]. FAAR’s main declared aim is “to fight drunk driving and underage drinking and to promote responsible decision-making regarding beverage alcohol.” [41]. Such CSR activities had originally been located within TAs prior to the advent of dedicated organisations [42]. FAAR and DISCUS have overlapping personnel; the President and CEO of DISCUS now serves as the head of FAAR and several other key leadership roles have shared responsibilities across both organisations. FAAR has nonetheless been retained as a separate organisational brand [43]. The rationale for more tightly integrating these organisations is unclear.

### Framing policy debates

DISCUS is active in shaping how policymakers approach alcohol as a policy issue at the domestic and international levels. Framing is distinct from lobbying (see below) in that it aspires to narrow decision makers’ attention toward a specific dimension of a policy issue, with a view to managing salience [44, 45].

At the domestic level, DISCUS has relied upon framing to advance its policy goals. First, the economic dimensions of alcohol, as opposed to health and social costs considerations, are regularly emphasised in particular ways. DISCUS actively highlights the economic costs borne by the sector during policy debates:

[t]he distilled spirits industry faces legislative and regulatory challenges that impact responsible adult consumers, including punitive taxes, international trade barriers and tariffs, and restrictions on consumer convenience [46].

DISCUS materials make little mention of the public health or social costs and consequences of the use of its products. Moreover, in its communications with lawmakers, the organisation regularly cites the contribution of the industry to the state or local economy:

[The spirits industry] is a major contributor to the state of Maryland, generating nearly $2.3 billion in economic activity and $292 million to local communities and the state in taxes [47].

DISCUS regularly emphasises the well-being of smaller producers and retailers in its advocacy efforts. For example, in a recent debate over reducing federal taxes on spirits producers, DISCUS claimed:

This… critical piece of legislation… will protect jobs, boost communities and get these small businesses back on a path of stability and growth… The reduced tax rates… will serve as an economic lifeline for beleaguered small distilleries that have had their tasting rooms shut down for months [48].

DISCUS is adroit at linking frames and issues. For example, there was a recent debate over whether the United States Postal Service (USPS) should be allowed to deliver alcohol. In making its case for supporting this legislation, small producers are identified as the main beneficiary of the policy change:

This legislation would… support producers that have struggled during the COVID-19 health pandemic and government-mandated business closures and/or limits… these producers have experienced dramatic declines in revenue as fewer Americans go out for dinner or a drink, gather for events, or travel [49].

The language used in its communications appears highly consistent and is preserved when working in coalitions, giving particular priority to focusing specifically on the need to help small businesses. For example, in a recent letter to Congress, DISCUS and its allies write:

Due to the extreme economic duress brought on by the COVID-19 pandemic, [small] businesses have been devastated. Small beverage alcohol producers have seen average revenue losses of 40% or more. We are asking you to support the alcohol beverage producers in your state and congressional district by co-sponsoring [this] legislation [50].

The rhetorical power of such material works to elide the interests of the large companies who run DISCUS and who will benefit from such legislation. DISCUS has also used COVID-19 as an opportunity to present itself as a good corporate citizen in other ways. DISCUS, as did companies and TAs elsewhere, regularly pointed out that there were over 800 distilleries in the US that were producing hand sanitiser for first responders and health care facilities [51]. The organisation also launched an online portal to connect distillers with industry suppliers and distribution channels [52].

DISCUS has a long history of operating at the international level [6]. DISCUS has influenced recent debates about alcohol policy, for example, through submissions to the World Health Organization (WHO) in 2018 and 2020. The framing work therein is consistent with other industry efforts to shape policymaking at the national and international level [22, 53]. First, DISCUS seeks to re-frame alcohol as a policy problem by moving the emphasis away from the societal costs or population-level harms and focusing on alcohol consumption among specific subgroups, by claiming that most people drink unproblematically:

[Most] adults who consume alcohol do so responsibly and in moderation. Consequently, the Commission’s recommendations for governments to explore policies to minimize production, marketing and consumption are misguided and do not accomplish the goals of the Global strategy to reduce the harmful use of alcohol.

Second, DISCUS makes unsubstantiated claims about the effectiveness of population-level interventions, pointing to limitations or perceived flaws with the existing literature, regardless of the actual content of that literature:

By focusing on the ‘best buys,’ [the WHO] ignores the scientific literature demonstrating the ineffectiveness and variability of these measures in reducing harmful alcohol consumption and failing to appropriately consider the effectiveness of other interventions [54].

Similarly, DISCUS points to the industry’s long preferred policy approaches, using targeted approaches to address “harmful drinking” rather than whole population approaches such as increasing price, reducing availability and marketing, and promoting education programs and family-based interventions instead [55].

Finally, DISCUS positions itself and other industry groups as legitimate participants in the international public health policymaking process. For example, according to DISCUS:

The U.S. private sector is an important contributor to evidence-based policymaking at WHO and in other fora. Private sector engagement is and has been effective in government efforts to address pressing health issues by providing additional resources, evidence-based measures and technical collaboration to implement sound, effective policies [54].

Again, such claims are advanced in strong terms, and notwithstanding the actual evidence, which is rather different. Moreover, in responding to the WHO’s working document in 2020, DISCUS complained:

The [WHO] takes an unduly negative view of engagement with economic operators… sound, effective efforts to address harmful use of alcohol require evidence-based measures and technical collaboration across the whole of society, including with economic operators [55].

### Lobbying

As the leading US group representing the interests of the spirits industry, engagement with federal and state officials, represents a key function of DISCUS. This study provides insights into the nature and scale of DISCUS’s lobbying efforts.

#### How much money does DISCUS spend on lobbying?

DISCUS’s materials indicate that federal lobbying is a major component of its public affairs strategy [35] but the organisation’s website does not specify how much money it allocates to this activity. In 2021, DISCUS spent more than $4.5 million on federal lobbying, according to data collected from OpenSecrets. Table 2 reports total expenditures for major alcohol industry actors in the United States for the most recent year available, above a minimum threshold of $500,000. DISCUS was only second to AB InBev in total lobbying expenditures for 2021, without including FAAR, and both entities greatly exceeded all others in expenditure, amongst which DISCUS member companies are also prominent. Between 2008 and 2021, its lobbying expenditures ranged between $4–6 million, with an average annual spend of about $5 million according to OpenSecrets.

**Table 2:** Federal lobbying expenditures among beer, wine & liquor industry groups (2021)

Client/Parent	Total (USD)
Anheuser-Busch InBev	$5,040,000
Distilled Spirits Council of the US (DISCUS)	$4,570,000
Molson Coors Brewing	$2,840,000
Diageo PLC	$2,710,000
Beer Institute	$2,660,000
Pernod Ricard	$2,380,000
Brown-Forman Corp	$1,200,000
Suntory Holdings	$1,110,000
Wine & Spirits Wholesalers of America	$1,020,000
National Beer Wholesalers Association	$850,000
Vineyard Wind	$630,000
Bacardi Ltd	$590,000
Constellation Brands	$560,000
Foundation for Advancing Alcohol Responsibility (FAAR)	$521,389
L’arche Green NV	$500,000

**Fig. 1:**
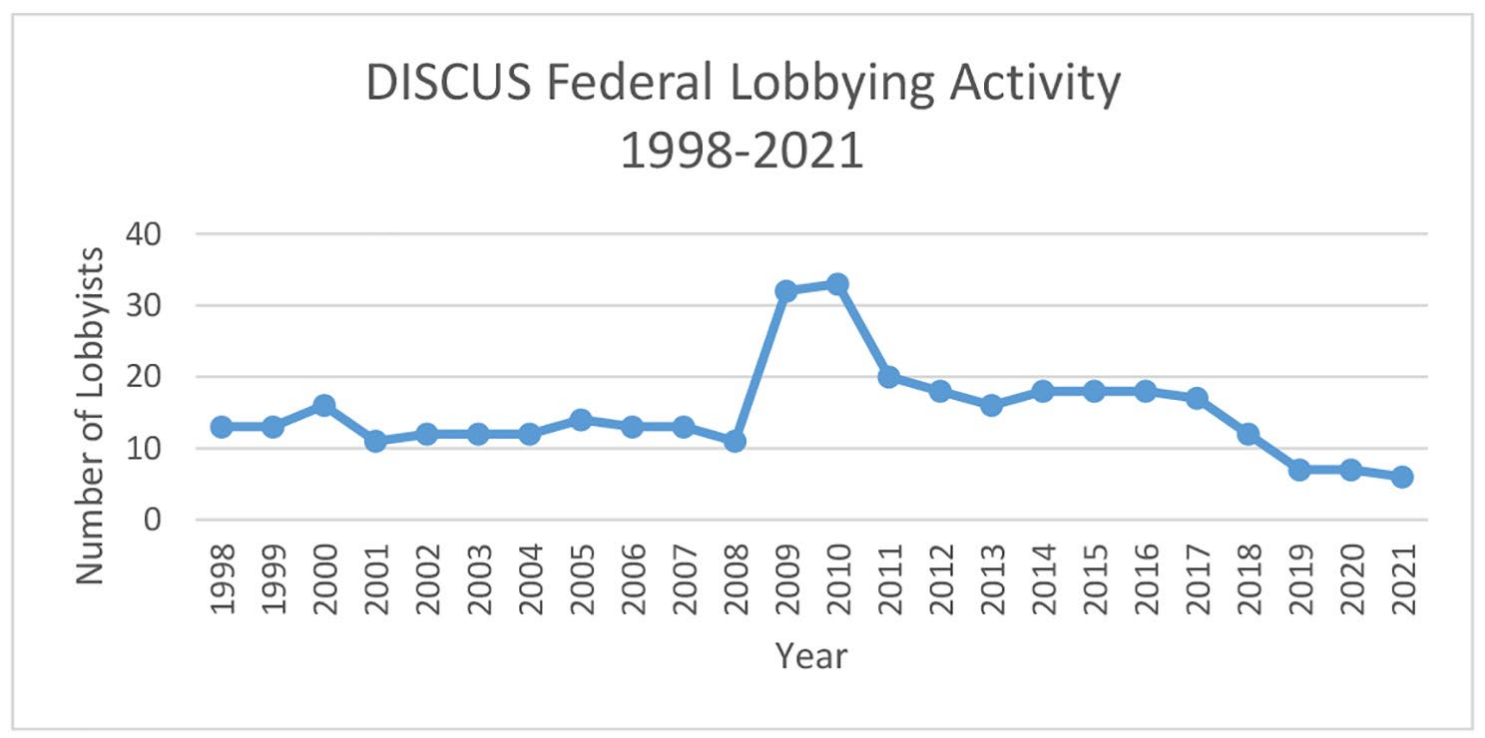
DISCUS Federal Lobbying Activity, 1998-2021

Figure 1 provides some additional insight into the scale of DISCUS’s lobbying operation. In recent years, DISCUS has employed around 7 lobbyists. Previously, however, this figure was much higher, particularly in 2009 and 2010, presumably relating to specific legislation under consideration at the federal level, and perhaps also contributing to US involvement in the 2010 WHO Global strategy process. As Table 2 shows, other organisations have lobbying interests which overlap with those of DISCUS, meaning the total number of alcohol industry lobbyists active at the federal level will be much higher.

DISCUS has also sought to gain influence through campaign finance activities. These include the formation of a Political Action Committee (PAC). PACs are organisations that enable members to pool campaign contributions and use these funds to finance campaigns for specific candidates, ballot initiatives or legislation. Campaign finance laws require PACs to disclose information about donors as well as the specific activities that are being financed [56]. DISCUS created the Distilled Spirits Council of the United States Inc. Political Action Committee (DISPAC). As well as to federal candidates, DISPAC makes contributions to candidates at the local and state level [57].

According to FEC filings, several of the DISPAC’s regular donors are DISCUS senior managers, including the CEO, senior vice presidents, and vice presidents [56, 58, 59]. Older FEC records reveal that several of the most significant contributions to DISPAC came from employees of DISCUS member companies, including Pernod Ricard USA [60], Brown Forman [61] and Patron Spirits Company [60]. Campaign contributions operate at a much lower level of expenditure than lobbying, with DISPAC’s spending averaging at just under $78,000 per election cycle.

#### What are some of the main targets of DISCUS’s lobbying efforts?

In the US, alcohol policies are not just established by Congress but are also subject to regulatory decisions made by federal executive departments and agencies. Between 1998 and 2021, DISCUS’s most common targets were the Departments of Agriculture, Commerce, Defence, Health and Human Services, State, Treasury, the Federal Trade Commission, the US Trade Representative, and the White House [62–84]. The responsibilities of these departments are largely in line with DISUS’s stated policy priorities (e.g., taxes, trade), though engagement with Health and Human Services is noteworthy.

Between 1998 and 2021, DISCUS lobbied federal officials on several issues according to OpenSecrets. The policy issues that are identified most frequently include taxes, alcohol & drug abuse, trade, agriculture, and advertising (for example, see [85, 86].

DISCUS materials offer a more restricted view of the issues it is focused on at the federal level. Much of DISCUS’s press releases and documents identify trade issues, particularly tariffs on distilled spirits as key policy priorities. Over the last few years, DISCUS has been highly focused on the removal of all tariffs on spirits [51]. Another key federal target is that taxes on ready-to-drink (RTD) beverages be taxed at the same rate since spirit-based RTDs have long been taxed at a higher rate than beer and wine [87].

DISCUS identifies state-level lobbying or “advocacy” as a key activity, perhaps unsurprisingly as state governments hold legal authority over many policy issues that affect the production and sales of spirits. DISCUS identifies “state market modernization” as one of its main areas of focus. In practice, this has meant lobbying for the reduction of taxes on spirits products and eliminating restrictions on alcohol sales [88]. Numerous states, for instance, have bans on spirits sales on Sundays. According to one briefing note, one of DISCUS’s main legislative priorities for 2022 was to promote the repeal of these bans [89]. DISCUS’s lobbying efforts have been successful in removing Sunday sales restrictions in Oregon [90] and North Carolina [91].

DISCUS has made liberalising alcohol delivery laws a major legislative priority, particularly in the context of the COVID-19 pandemic. When public health restrictions were first introduced in March 2020, some states began allowing restaurants and bars to sell cocktails to-go. The State of New York’s decision to temporarily allow cocktail delivery quickly diffused across the US, with 35 states following New York’s lead [92]. DISCUS continues to invest heavily in lobbying state governments to make these arrangements permanent. On cocktail delivery alone, we collected over 100 relevant press releases and other documents (e.g., witness testimony to legislative committees). Since 2021, a total of 18 states have enacted permanent cocktails to-go legislation, with DISCUS having been involved in lobbying in all but two. During a presentation to staff and DISCUS members in February 2022, CEO Chris Swonger hailed these policy changes as some of the organisation’s most important legislative “victories” [89].

#### How has DISCUS used mass political mobilisation to enhance its lobbying efforts?

DISCUS has also used mass political mobilisation to advance political influence. In 2019, DISCUS created the Spirits United campaign. Spirits United is a joint initiative with the American Distilling Institute, and several state-level TAs [93]. It is described as a “grassroots platform” aimed at bringing together spirits advocates, including spirits professionals and consumers. Website visitors are told that “by joining Spirits United, you can make an impact by making your voice heard on key issues facing the distilled spirits industry”[94]. The Spirits United platform is designed so that consumers can contact their lawmakers about spirits-related legislation. Visitors are asked to supply their personal information, including their home address, which is then used to facilitate correspondence between individual members and elected officials [95]. Spirits United is focused on mobilising the public to influence a range of policy debates. These include federal spirit taxes and tariffs (particularly in the context of free trade agreements), increased availability of alcohol, reducing regulations on spirits tastings, and reducing taxes on spirits [96, 97]. Spirits United is seen as a particularly important resource for state-level efforts. As one campaign document explained:

Last year alone, over 2,000 bills related to beverage alcohol were introduced in the states, including proposals to increase spirits tax rates in 18 states. [DISCUS] will be utilizing Spirits United in support of our efforts to defend against taxes and improve market access for spirits in the states! [97]

Unlike traditional lobbying, Spirits United appears modelled on a different conception of how to build political support for the spirits industry’s policy goals. DISCUS materials suggest that mobilising consumers and other indirect stakeholders is a key tactic for securing industry goals. According to one presentation:

We are all competing for lawmakers’ attention. These [other] industries are taking advantage of a communication channel that our industry is not. We need individual engagement from everyone in the industry to ensure our messaging, needs, and concerns are at the top of Congress’ mind [97].

There is some evidence of successful political mobilisation. Between 2019 and 2020, the Spirits United platform led to “more than 65,000 communications to Congress” in support of the Craft Beverage Modernization and Tax Reform Act [98].

#### How has DISCUS used coalition-building to strengthen its lobbying operations?

DISCUS has also worked with other industry partners to achieve common goals, including creating the Hospitality Recovery Coalition (HRC), a partnership formed between DISCUS, the American Distilled Spirits Alliance (ADSA), the Council of State Restaurant Associations (CSRA), the National Restaurant Association and Training for Intervention Procedures (TIPs). The coalition’s aim is to “advocate for policies supporting on-premise establishments facing financial turmoil due to the COVID-19 crisis”[99]. HRC engages in several policy-influencing efforts, including lobbying state leaders and lawmakers to make cocktails to-go legislation permanent [100].

During the pandemic, DISCUS also worked with a broad coalition across the hospitality sector to promote relief for small businesses. This included the Independent Restaurant, American Beverage Licensees, American Cider Association, Beer Institute, Brewers Association, National Restaurant Association, Produce Marketing Association, Wine America, Wine Institute, and Wine & Spirits Wholesalers of America [49]. Creating large coalitions that cut across different parts of the alcohol sector allows DISCUS and other corporate interests to claim that they represent a larger segment of the American economy.

DISCUS has used similar coalition-building tactics in promoting the passage of a range of bills at the federal level, including RESTAURANTS Act of 2021 (S. 255/ H.R. 793), the Fairness for Craft Beverage Producers Act (H.R. 1035) and the Hospitality and Commerce Job Recovery Act of 2021 (H.R. 1346/ S.477). DISCUS has promoted support for this legislation alongside the Beer Institute and Wine Institute, the other key alcohol TAs in the U.S.

## Discussion

This case study advances understanding of DISCUS as a political actor and provides new insights into the policy roles of this alcohol TA that may be generalizable to other alcohol actors and other sectors. Although in an important sense preliminary, it is striking just how much of the activity of DISCUS is captured in this analysis of lobbying and framing. This in turn may contribute to enhanced understanding of the coordinated nature of the broader alcohol industry’s political strategy.

Among several novel insights into the nature of TAs as political organisations, this study uncovers similarities and differences between alcohol and tobacco TAs. DISCUS was established to communicate the spirit industry’s policy positions to policymakers and to coordinate public relations campaigns. Before it was disbanded in the Master Settlement Agreement (MSA), the Tobacco Institute, the tobacco industry’s main TA in the US, was created for a similar purpose [14]. There is also overlap in the tactics employed by TAs in both sectors, particularly lobbying, campaign contributions, public relations campaigns, and alliance building [101], as well as in leadership [6]. Key differences are also apparent. DISCUS strives to be a politically visible policy participant, actively engaging with the media. In contrast, since the MSA, tobacco companies have routinely used front groups or “astroturf” [102] organisations to be the public face of their various political campaigns and operations [101, 103, 104]. This strategy is used to remove the tobacco industry’s “fingerprints” from such campaigns [105, 106]. The differences in circumstances allow DISCUS to pursue distinct ways of shaping public understanding of its products and of the alcohol industry, including high-profile CSR activities. This study also provides insights into how this alcohol TA used major shifts in the economic and political environment to advance its interests. In 2020, with the onset of the COVID-19 pandemic, concerns about the viability of small businesses were prominent in political discussions. DISCUS seized this opportunity and presented alcohol liberalisation as a policy solution, arguing these policies would provide a “lifeline” for restaurants and small distilleries. DISCUS’s lobbying efforts were critical in generating an unprecedented surge of alcohol policy making activity at the state level. DISCUS’s response to the hand sanitiser shortage is another key example, and one which was common to large alcohol companies and TAs elsewhere. DISCUS highlighted the distiller’s CSR activities to the press and the public. Efforts of this nature might not seem significant but these can have important, yet subtle, effects on public perceptions of the alcohol industry. These provide an opportunity for the industry to cast themselves as good corporate citizens and legitimate policy participants in advancing public health priorities [1]. Our analysis of DISCUS, then, demonstrates the nimble and sophisticated quality of alcohol TA’s political tactics. This finding is consistent with other studies which stress that moments of crisis generate opportunities to be exploited by well-organised groups [107–110].

Building on previous work on the politics of alcohol policy [2, 33, 111–120], the present study demonstrates how the concept of framing is also useful for analysing the alcohol industry’s political influence [2]. Attention to framing allows researchers to better understand the content of a political actor’s goals but also could enable assessment of the industry’s influence on policy (i.e., framing effects). Our study suggests that framing can be partially about narrowing down the group of salient stakeholders. For example, we show how DISCUS disproportionately focused on small producers and retailers. This is not necessarily a novel political strategy. As noted above, large businesses, including alcohol and tobacco companies, have often allied themselves with groups that are more likely to elicit public sympathy [121–123]. How the alcohol industry is understood, as well as how alcohol itself is understood by the public and policy actors is important to distinguish.

Previous studies of the alcohol industry’s political tactics, including lobbying and political donations [118, 124–127] rarely specify the individual targets of lobbying, as done here. As the lobbying expenditure data show, DISCUS is one among many alcohol industry groups active in Washington DC [10, 128]. Moreover, DISCUS’s public-facing materials rarely make reference to the health and social-related impacts of alcohol but our findings reveal that the Department of Health and Human Services is one of the organisation’s most common lobbying targets. Although the precise nature of these discussions and the effectiveness of these lobbying efforts is unclear from the data, this suggests that health-related issues, or perhaps the framing of these issues for policymakers, are a major priority for distilled spirits, and likely other sectors of the alcohol industry. To develop our understanding of the effectiveness and scope of such lobbying, we will require studies dedicated to the activities of the various players working together [33]. Similarly, although we have separated them for analytic purposes, we draw attention here to the clear inter-dependencies and synergies between framing and lobbying strategies, a theme identified in previous work [119].

Moreover, additional comparative research is needed to examine the operations and functions of other TAs in the US and beyond. There is a broader literature on the functions of trade associations in other contexts and particularly their roles in coordinating and leading CSR activities [5, 129]. Historical research on asbestos, for example, shows how TAs can shape regulatory responses to public health issues through strategic framing of scientific evidence in particular [18]. In the case of alcohol, there is growing evidence of the industry seeking to shape scientific debates on alcohol-related harm [23, 32, 130]. This is a long established and strategically vital practice for the US distilled spirits sector, dating back to its predecessor organizations in the 1950s, before DISCUS was formed in 1973[6]. The role of DISCUS and other such actors internationally, and at the global level, requires further study.

The study has several limitations worth considering. First, it relies entirely on documents that are in the public domain and thus cannot be expected to entirely capture how DISCUS really works internally, and how it understands and develops its strategic goals and activities over time. Achieving such understanding would require internal documents and/or interviews with present or former DISCUS employees. Moreover, interview data introduces some reliability issues which must also be considered. A more feasible approach might be to conduct interviews with some of the government officials and other actors that DISCUS regularly engages with to discuss alcohol policy. Second, some of the other sources analysed for this study have limitations that need to be recognised in what they can reveal about DISCUS and TAs more generally. For example, lobbying expenditure data is helpful for illustrating the resources (e.g., people and money) allocated to an activity but it cannot capture the extent to which these efforts are successful. Third, the scale and detail of the individual lobbying records meant that this study did not provide an in-depth analysis of all the legislation and policy issues that DISCUS has lobbied the federal government on. Future work should consider these individual records as a key resource for data triangulation purposes. For example, in seeking to understand a TA’s efforts to shape a specific piece of legislation, researchers might cross-reference claims from research interviews with the details of individual lobbying records. Fourth, there are a set of limitations to be considered that are contingent upon the novelty of the study for alcohol, which in itself is indicative of a major contribution of this study. Because the study of the alcohol industry is under-developed in comparison with other health-harming corporate sectors such as tobacco or food, we are not in a position to locate DISCUS strategically within the nexus of companies, CSR organisations and other TAs that constitute the alcohol industry. As a consequence the contribution of an in-depth study of DISCUS makes a necessarily limited contribution to the literature on TAs in other corporate sectors. Finally, a strength of the study is that it relies on a range of data sources to study DISCUS. For example, the analysis of press releases and other documents gives insights into the range of policy issues being prioritised and monitored by the alcohol industry.

## Conclusions

A growing body of work points to the politically sophisticated nature of the alcohol industry and its efforts to shape science and policy [2, 10, 13, 20, 23, 44, 128, 131, 132]. There is further work to do to identify the ways in which industry actors think about and engage with domestic and international political processes. Analysing materials authored by alcohol industry actors offers a key resource for studying the nature of these organisations. Future research should examine other materials that exist in the public domain, including annual reports, tax records, and other mandatory disclosures. This research study, whilst modest in design, demonstrates what can be accomplished by analysing publically available data. To generate more secure inferences about the nature of the alcohol industry’s efforts to advance its interests, and with what success and at what cost, researchers will need to investigate other TAs in different contexts, and use other data sources.

## Data Availability

All data generated or analysed during this study are included in this published article or in the hyperlinks provided.

## Footnotes

1 Retrieved 3 March – 4 March 2022.

2 Retrieved 21 April 2022.

3 Retrieved 26 January 2022.

4 Retrieved 26 January – 28 January 2022.

5 Retrieved 18 April 2022.

6 Retrieved 18 April 2022.

7 Retrieved 18 April 2022.

8 Retrieved 5 May 2022.

## List of Abbreviations

ADSA: American Distilled Spirits Alliance
CSR: Corporate Social Responsibility
CSRA: Council of State Restaurant Associations
DISCUS: Distilled Spirits Council of the United States
DISPAC: Distilled Spirits Council of the United States Inc. Political Action Committee
FEC: Federal Election Commission
FAAR: Foundation for Advancing Alcohol Responsibility
MSA: Master Settlement Agreement
PAC: Political Action Committee
RTD: Ready-to-drink
SOPR: Senate’s Office of Public Records
TA: Trade association (TA)
US: United States
USPS: United States Postal Service
WHO: World Health Organization
